# A Free-Energy Landscape Analysis of Calmodulin Obtained from an NMR Data-Utilized Multi-Scale Divide-and-Conquer Molecular Dynamics Simulation

**DOI:** 10.3390/life11111241

**Published:** 2021-11-16

**Authors:** Hiromitsu Shimoyama, Yasuteru Shigeta

**Affiliations:** Center for Computational Sciences, Unviersity of Tsukuba, 1-1-1 Tennodai, Tsukuba 305-8577, Japan

**Keywords:** molecular dynamics simulation, calmodulin, domain motion, dynamics, conformational change, free-energy analysis

## Abstract

Calmodulin (CaM) is a multifunctional calcium-binding protein, which regulates a variety of biochemical processes. CaM acts through its conformational changes and complex formation with its target enzymes. CaM consists of two globular domains (N-lobe and C-lobe) linked by an extended linker region. Upon calcium binding, the N-lobe and C-lobe undergo local conformational changes, followed by a major conformational change of the entire CaM to wrap the target enzyme. However, the regulation mechanisms, such as allosteric interactions, which regulate the large structural changes, are still unclear. In order to investigate the series of structural changes, the free-energy landscape of CaM was obtained by multi-scale divide-and-conquer molecular dynamics (MSDC-MD). The resultant free-energy landscape (FEL) shows that the Ca^2+^ bound CaM (holo-CaM) would take an experimentally famous elongated structure, which can be formed in the early stage of structural change, by breaking the inter-domain interactions. The FEL also shows that important interactions complete the structural change from the elongated structure to the ring-like structure. In addition, the FEL might give a guiding principle to predict mutational sites in CaM. In this study, it was demonstrated that the movement process of macroscopic variables on the FEL may be diffusive to some extent, and then, the MSDC-MD is suitable to the parallel computation.

## 1. Introduction

Calcium ions (Ca^2+^) act as intracellular messengers that relay information within cells to regulate cellular processes [1,2]. The information of a transient Ca^2+^ signal is converted into a wide variety of biochemical changes by Ca^2+^-binding proteins (also known as Ca^2+^-sensors) through Ca^2+^-induced conformational changes [3,4]. Calmodulin (CaM) is a ubiquitous Ca^2+^-sensor found in eukaryotic cells, which activates enzymes, such as kinases and phosphatases, in a Ca^2+^-dependent manner so that it regulates various biochemical processes. In the early 1970s, the activation activity by CaM was discovered in a study of phosphodiesterase (PDE), and then its activating factor was identified as a Ca^2+^-binding protein [5,6,7,8,9]. Nowadays, CaM is known to be involved in various cellular processes, such as cell proliferation, apoptosis, muscle contraction, inflammations, immune responses, and long-term potentiation [3,4,10,11,12,13,14,15,16,17,18].

CaM consists of 148 residues and it has four special motifs called ‘EF-hand’; the EF-hand specifically interact with Ca^2+^. The reason is that the EF-hand sequence contains a highly conserved motif at the binding sites; the motif is the most common motif found in Ca^2+^-binding proteins [4,19,20,21,22]. The EF-hand motifs have been found in other Ca^2+^-binding proteins, such as Troponin C and Calbindin, and these proteins comprise a “EF-hand protein family” [19,20,21,23,24]. In the solution state, The EF-hand motif forms a helix-loop-helix structure [19,20,25,26,27]. The canonical EF-hand has the central loop consisting of many glutamates and aspartates (negatively charged amino acids), and then these amino acids encourage the loop to capture Ca^2+^ ions [4,20,21].

Monomeric structures of both Ca^2+^-bound CaM (holo-CaM) [28,29,30,31,32,33,34,35,36,37] and unbound CaM (apo-CaM) [38,39] have been studied experimentally. For example, X-ray crystallography and nuclear magnetic resonance (NMR) experiments have shown that CaM has two almost symmetrical globular domains; the domains are separated by a long linker region. The 1st and 2nd EF-hand are contained in the N-terminal domain (N-lobe), and the 3rd and 4th EF-hand are contained in the C-terminal domain (C-lobe). The structures of the N-, C-lobe, and linker region of apo-CaM are different from those of holo-CaM, indicating that CaM undergoes conformational change induced by Ca^2+^-binding.

Both N- and C-lobe have four helices (the entering- and exiting-helix of two EF-hands). The NMR experiments have shown that the helices in apo-CaM form a single twisted four-helix-bundle. The linker region is disordered and flexibly connects the N-lobe and C-lobe, and then, the N- and C-lobe are expected to almost freely move independently from each other in the solution [38,39]. On the other hand, when Ca^2+^ binds, the EF-loop is pinched by Ca^2+^ and the interaction between the entering- and exiting-helix of each EF-hand is inhibited, causing the helices to separate each other [28,29,31,32,33,34,35,36,37]. This structural change exposes hydrophobic amino acids buried in the helix bundle, forming a hydrophobic pocket; the pocket is the binding site for the target protein [19,20]. The EF-hand conformations of holo-CaM and apo-CaM are referred to as “open conformation” and “closed conformation”, respectively. In the holo-CaM structure, the linker region is often a continuous helix and the whole structure is dumbbell-shaped.

Ca^2+^-activated CaM regulates CaM-dependent proteins by forming a complex with them. Ordinary CaM-dependent proteins contain a CaM-binding domain (CaMBD), which is characterized by a high helix tendency and a net positive charge in the binding region. The structure of the CaM-CaMBD complex has been studied by X-ray crystallography and NMR experiments. For human CaM, the following complex structures with various proteins are known; CaM-dependent protein kinases (CaMK) [40,41], myosin light chain kinases (MLCK) [42,43,44], calcineurin (CN) [45,46,47,48], death-associated protein kinases (DAPK) [49,50], anthrax edema factor (EF) [51,52,53,54], and nitric oxide synthases (NOS) [55,56,57,58] have been deposited in the Protein Data Bank (PDB). In the canonical binding complex, the linker region is bent so that CaM wraps around CaMBD on the target protein. In 2013, Tidow et al. classified such canonical complexes with respect to their interaction patterns and reported conformational diversity of CaM-CaMBD complex [59].

In the field of biochemistry, a protein is often enhanced or reduced by an effector molecule that binds to a site distant from an active site [60,61]. Such phenomena are referred to as ‘allosteric regulation’ or ‘allostery’, and a molecule-enhancing activity is also called an ‘allosteric activator’, whereas a reducing one is called an ‘allosteric inhibitor’. In many proteins, the effector-induced signal propagation is closely associated with structural changes. The allostery is first attributed to the functional enhancement of hemoglobin in 1904, and today, the allostery is widely accepted as an essential mechanism of cellular processes [62,63]. For CaM, the Ca^2+^-binding site is spatially too distant from the linker region to directly interact with each other. Then, the allosteric regulation is expected to mediate in order to propagate the Ca^2+^-induced signal into an enzymatic activity. However, it is not yet clear how Ca^2+^ regulates the overall structure of CaM.

In 2008, Gsponer et al. reported structural ensembles representing the dynamics of CaM in a monomeric Ca^2+^-bound state (PDB ID 2k0e) and in complex with MLCK (PDB ID 2k0f) [43]. The structures of 2k0e show large diffusion-like movement, as well as that of apo-CaM (PDB ID 1dmo [38]); it is in contrast to the holo-CaM structures determined by X-ray crystallography, such as PDB ID 1cll [28] and 3cln [29]. Their results indicated that Ca^2+^-binding induces “population shift” through structural fluctuations, which gives rise to correlated motions. In our previous work, the structural ensemble reported by Gsponer (PDB ID 2k0e) was analyzed to identify the allosteric interactions [64].

Since the molecular dynamics (MD) simulation of CaM was performed by Komeiji et al. in 2002, many works employing the MD simulations have been made on CaM [65]. However, the SAXS experiment conducted in 2012 reported that the structural change of CaM takes from a millisecond to a second in a timescale [66]. The timescale currently affordable by ordinary MD simulation is a few hundred nanoseconds to microseconds, while the timescale of CaM is longer by more than 10^3^ times. Useful but time-consuming calculations, such as the calculation of the free-energy landscape (FEL), for example, are not possible with the conventional MD simulations.

To overcome the time limitation, many simulation methods that facilitate effective sampling, have been proposed. For example, “umbrella sampling” that uses a harmonic potential or modified “flat-bottom” potential to facilitate an extensive sampling, is widely used [67,68,69]. Since the dimension of the conformation space is too large to sweep, collective variables, such as center-of-mass and RMSD, have been used to sweep multidimensional conformation space by lower dimensional sampling [70,71]. In our sampling, variables from principal component analysis (PCA) were used; the variables were chosen so that they were not affected by rotation and translation.

In a previous work, we proposed a method which combines an efficiency of a coarse-grained model (CGM) for conformational search and accuracy of an all-atom model (AAM) named multi-scale divide-and-conquer MD (MSDC-MD) [72] and demonstrated that the FEL of an intrinsically disordered protein, which is one of CaMBDs, could be evaluated correctly. In the present work, MSDC-MD was applied to the large conformational change of CaM by using the NMR experimental structures instead of CGM-MD-derived structures adopted before, and analyzed the FEL to obtain useful information for conformational change of CaM.

## 2. Materials and Methods

**Figure 1:**
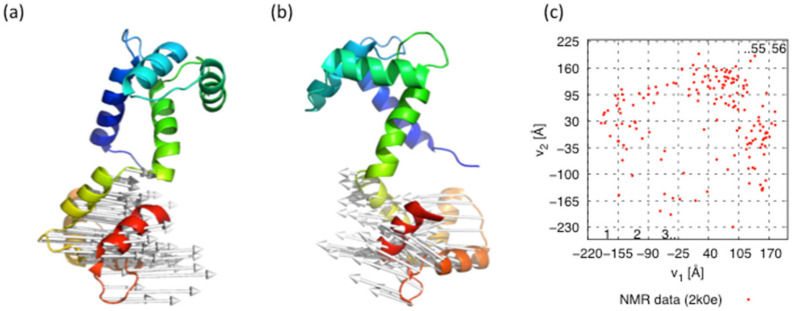
Porcupine plots of (**a**) the first eigenvector and (**b**) the second eigenvector mapped on the 20th model in NMR structures (PDB ID 2k0e). (**c**) Scatter plot of the NMR structures (PDB ID 2k0e). The *x*- and *y*-axis are the first and second principal component, respectively. The numbers of small areas are written sequentially.

In our previous work [64], the monomeric CaM structural ensemble (PDB ID 2k0e) was analyzed by PCA. The first and second principal components corresponded to the relative domain motion (Figure 1a,b), and the cumulative proportion of the variance was about 80%. The distribution of monomeric structures on the plane by the first and second principal components had a horseshoe shape, as shown in Figure 1c (such distribution was also reported by Kurkcuoglu et al. in 2016 [73]).

The NMR structures (PDB ID 2k0e) were analyzed by the PCA to determine the reaction coordinate. Before PCA, to remove the effects of translation and rotation, root-mean-square-distance (RMSD) fitting with respect to the “reference coordinate” (first model’s N-lobe, Cα positions 5-70; $s_{\nu }=\left(x_{\nu },y_{\nu },z_{\nu }\right)$ for ν = 5⋯70) was performed on the original Cα position $r_{i}=\left(x_{i},y_{i},z_{i}\right)$,

$$\begin{array}{ccc} r\prime_{i}=R\left(r_{i}-Q\right) & \mathrm{for} & i=1\cdots 148 \end{array}$$

$$Q=\frac{1}{66}\sum_{\nu =5}^{70}r_{\nu }$$

$$R={\left(A^{t}A\right)}^{1/2}A^{-1}$$

where the vector Q is the centroid of the fitting Cα atoms and the matrix *R* is a 3 × 3 rotational matrix. The rotational matrix *R* depends on $r_{\nu }$ and $s_{\nu }$ through the cross-covariance matrix *A*, defined as follows [74],

$$A_{ab}=\sum_{\nu =5}^{70}\left(r_{\nu a}-Q_{a}\right)\left(s_{\nu b}-P_{b}\right)\;\mathrm{for}\;a,b=1\cdots 3$$

$$P=\frac{1}{66}\sum_{\nu =5}^{70}s_{\nu }$$

where the vector P is the centroid of the reference coordinate. Then, the fitted coordinate $X\prime =\left(r\prime_{1},\cdots ,r\prime_{N}\right)$ was analyzed by PCA to obtain the eigenvalues and eigenvectors (*N* = 148). The projection of the structure to *n*th principal component ($v_{n}$) can be written as $v_{n}=\sum_{k=1\cdots 3N}\;c^{\left(n\right)}{}_{k}\cdot X\prime_{k}$ where $c^{\left(n\right)}$ is the *n*th eigenvector of a variance-covariance matrix ${\left(\Lambda \right)}_{kl}=\langle \left(X\prime_{k}-\langle X\prime_{k}\rangle \right)\cdot \left(X\prime_{l}-\langle X\prime_{l}\rangle \right)\rangle$ of X′ (〈⋯〉 indicates an ensemble average, k,l=1⋯3N), which satisfies the following equation.

$$\Lambda c^{\left(n\right)}=\lambda_{n}\cdot c^{\left(n\right)}$$

**Figure 2:**
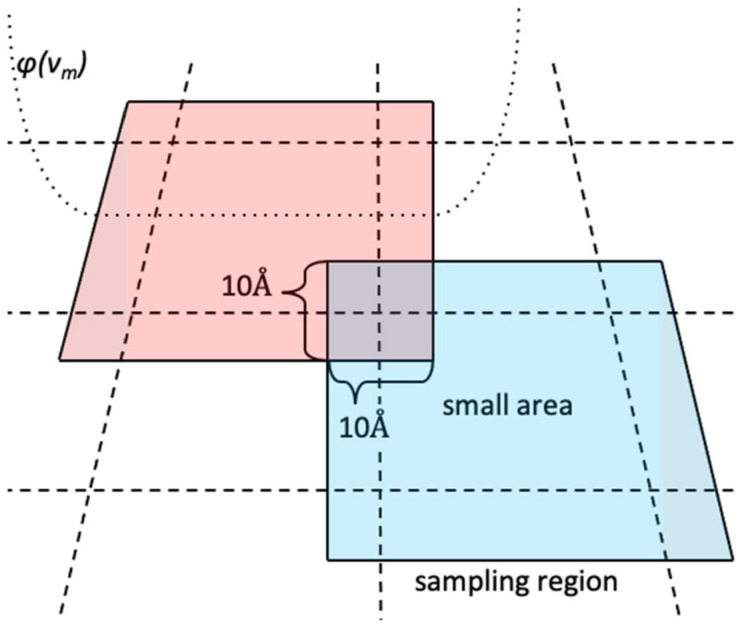
Graphical definition of the small area (dashed line), sampling region (solid line), and *ϕ*(***v***_m_) (dotted line).

In order to perform MSDC-MD, the monomeric structures on the $v_{1}$, $v_{2}$-plane were divided into 56 small areas (Figure 1c and Figure 2 dashed line), where the sizes of the small areas were chosen so that the following all-atom model (AAM) MD simulations can sweep the small area. Note here that this process is based on trial and error to date, because the way to know the optimum size of the area is unknown a priori. When the size is too large, individual AAM-MD cannot sweep the area, nevertheless, when the size is too small, the ergodicity might break down.

Multiple AAM-MD simulations were initiated at a randomly selected AAM structure using a modified version of GROMACS [75] (in a small area without the monomeric structure, AAM-MD simulation was not performed). To estimate the statistical error, this process was performed three times, changing the initial structure and the initial velocity. For the simulations, AMBER03 [76] force field was used for the protein and ions. Ca^2+^ parameter was σ = 3.0524 Å and ϵ = 0.45962 kcal/mol [77]. The periodic boundary condition in that 20 Å buffer was taken from the most outer atom, was used and CaM was solvated in the TIP3P water models [78]. The numbers of water molecules were different for different initial structures, and then the total number of atoms varied from 52,754 to 68,597. The particle mesh Ewald method [79,80,81], with cutoff = 10 Å, was used for Coulomb interaction calculations. The total charge of the system was neutralized by adding Na+ ions. The Lennard-Jones potential, with cutoff = 14 Å, was used for van der Waals interactions. After energy minimizations and 1 ns of equilibration simulations with position restraints, 200 ns of multiple production simulations were performed independently, and for all AAM-MD simulations, the time step was 2 fs. The temperature and pressure of these simulations were maintained to be *T* = 300 K and *P* = 1 bar by the Berendsen thermostat [82] and Berendsen barostat [83], respectively. In the production runs, the following umbrella-like potential was imposed.

ϕvm=k2vmmin−vm2forvm<vmmin0forvmmin≤vm≤vmmaxk2vm−vmmax2forvmmax<vm

where m=1 or 2, the spring constant $k=0.048\mathrm{kcal}/\mathrm{mol}/{Å}^{2}$, $v_{m}^{\min}$ and $v_{m}^{\max}$ are the lower and upper limits of the sampling region, respectively. The difference between “small area” and “sampling region” is illustrated in Figure 2. The sampling regions overlap with the neighboring small areas by 5 Å. For example, the parameters of the sampling region 1 and 2 are taken to be $v_{1}^{\max\left(1\right)}-v_{1}^{\min\left(2\right)}=10Å$. Then, the relative weight of between the small area 1 and 2 can be written as $\omega_{12}=\log\left(N_{2}\right)-\log\left(N_{1}\right)$, where $N_{1}$ and $N_{2}$ are the number of the samples from the sampling region 1 and 2 in $v_{1}^{\min\left(2\right)}\leq v_{1}\leq v_{1}^{\max\left(1\right)}$. In general, the relative weight between the small areas *i* and *j* can be written as $\omega_{ij}=\log\left(N_{j}\right)-\log\left(N_{i}\right)$, where $N_{i}$ and $N_{j}$ are the number of samples from the sampling region *i* and *j* in $v_{m}^{\min\left(j\right)}\leq v_{m}\leq v_{m}^{\max\left(i\right)}$. All structural samples in $v_{m}<v_{m}^{\min}$ or $v_{m}^{\max}<v_{m}$ were excluded from the FEL calculation. The entire weight over all sampling regions ($W_{i}$) is then obtained as follows.

$$W_{j}^{\left(c+1\right)}=\omega_{ij}+W_{i}^{\left(c\right)}\;\mathrm{for}\;N_{i}>0\;\mathrm{and}\;N_{j}>0$$

where *i,j* are indexes of the sampling region, *c* is the iteration number and $W_{\nu }^{\left(0\right)}=0$; the initial sampling region ν is determined so that the number of overlapping regions is maximized, as, even if there is no overlap with the next region, the relative weight might be determined through the diagonally across region. Then, the above iteration was continued until all regions were linked. The entire FEL was obtained by summing the samples in the sampling region *i* with the final weight $W_{i}$ as follows.

$$F\left(v_{1},v_{2}\right)=k_{B}T\;\ln\left[\frac{\sum_{i=1}^{56}\exp\left(W_{i}\right)\left\{\frac{\sum_{t}\delta \left(v_{1}\left(t\right)-v_{1}\right)\;\delta \left(v_{2}\left(t\right)-v_{2}\right)/M\left(v_{1}\left(t\right),v_{2}\left(t\right)\right)}{L_{i}}\right\}}{\sum_{i=1}^{56}\exp\left(W_{i}\right)\left\{\frac{\sum_{t}1/M\left(v_{1}\left(t\right),v_{2}\left(t\right)\right)}{L_{i}}\right\}}\right]$$

where $k_{B}$ is the Boltzmann constant, the index t and $L_{i}$ are, respectively, the time step and the number of samples obtained from each sampling region *i* (please remember that samples out of the sampling region is excluded) and $M\left(v_{1}\left(t\right),v_{2}\left(t\right)\right)$ is the number of overlapping; if $\left(v_{1}\left(t\right),v_{2}\left(t\right)\right)$ is included in a region that is swept twice, M = 2. For this study, the difference of the entire FEL caused by the different choice of ν was satisfactory smaller than the statistical error.

The force of $\phi \left(v_{m}\right)$ can be written as follows.

$$-\nabla \phi \left(v_{m}\right)=\left\{\begin{array}{ccc} k\left(v_{m}^{\min}-v_{m}\right)\nabla v_{m} & \mathrm{for} & v_{m}<v_{m}^{\min}\\ 0 & \mathrm{for} & v_{m}^{\min}\leq v_{m}\leq v_{m}^{\max}\\ k\left(v_{m}-v_{m}^{\max}\right)\nabla v_{m} & \mathrm{for} & v_{m}^{\max}<v_{m} \end{array}\right.$$

Since the vector Q and the rotation matrix *R* depend on original Cartesian coordinates $X_{i}=\left(r_{1},\cdots ,r_{N}\right)$, $\nabla v_{m}$ can be written as follows.

$$\begin{array}{ll} \frac{\partial v_{m}}{\partial X_{i}}=\sum_{j=1}^{N}\sum_{a=1}^{3}c_{3j+k}^{\left(m\right)}\frac{\partial {X^{\prime }}_{3j+a}}{\partial X_{i}} & \\ =\sum_{j=1}^{N}\sum_{a=1}^{3}c_{3j+a}^{\left(m\right)}\{\sum_{b=1}^{3}\frac{\partial R_{ab}}{\partial X_{i}} & \left(X_{3j+b}-Q_{b}\right)+R_{ab}\left(\delta_{i,3j+l}-Q_{b}\right)+R_{ab}\left(X_{3j+l}-\frac{\partial Q_{b}}{\partial X_{i}}\right)\} \end{array}$$

In order to show the efficiency of MSDC-MD’s sampling, the non-arbitrary structure of a given area on FEL is shown as a “representative” structure. The representative structure was defined as a centroid in RMSD. Let $\Delta_{ij}$ be the RMSD of structure *i* with respect to structure *j*, then the total distance between an *i* and others are measured as $\Delta_{i}=\sum_{j\neq i}\Delta_{ij}$. Then, the representative structure was defined as that with the smallest $\Delta_{i}$.

In our previous study [64], the allosteric interactions were identified as a contact probability. These interactions are also used to analyze the FEL. Suppose two residues, *i* and *j*, where a distance between any atom in residue *i* and another atom in residue *j* is less than 4.5 Å, the residues are identified as a contacting residue pair.

## 3. Results and Discussion

**Figure 3:**
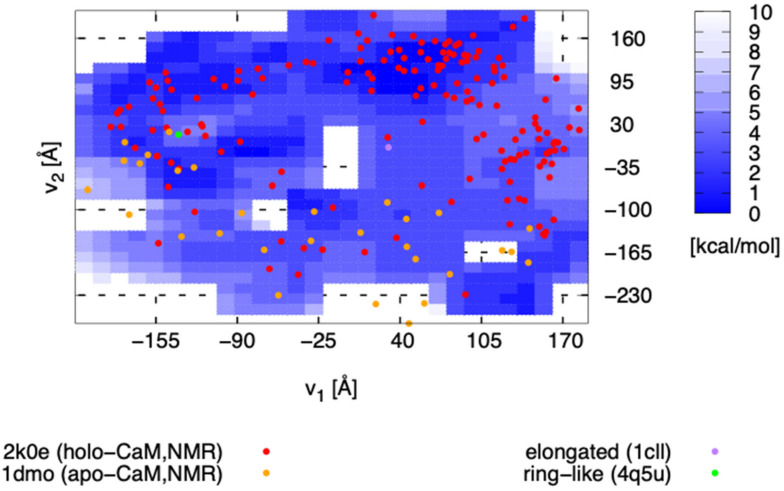
The entire free-energy landscape obtained from MSDC-MD. The *x*- and *y*-axis are respectively $v_{1}$ and $v_{2}$. The red and orange points show the holo-NMR and apo-NMR structures (PDB ID 2k0e, 1dmo), respectively, the green and purple points, respectively, show the elongated structure (PDB ID 1cll) and the ring-like structure (PDB ID 4q5u).

The entire FEL ($F\left(v_{1},v_{2}\right)$, T = 300 K, $k_{B}T$ = 0.6 kcal/mol) obtained by MSDC-MD is shown in Figure 3, which shows that the FEL has a complex multi-valley structure. The red points correspond to the NMR structures (Figure 3 red; PDB ID 2k0e), and it can be seen from Figure 3 that the distribution of the NMR structures roughly coincided with the basins of the FEL. The monomeric elongated structure (Figure 3 purple; PDB ID 1cll) is located at the edge of a broad basin around ($v_{1},v_{2}$) = (50, 100). Notably, the ring-like structure in the complex (Figure 3 green; PDB ID 4q5u) was not located in a stable FEL’s basin, but in a slightly higher free-energy (structurally unstable) region.

**Figure 4:**
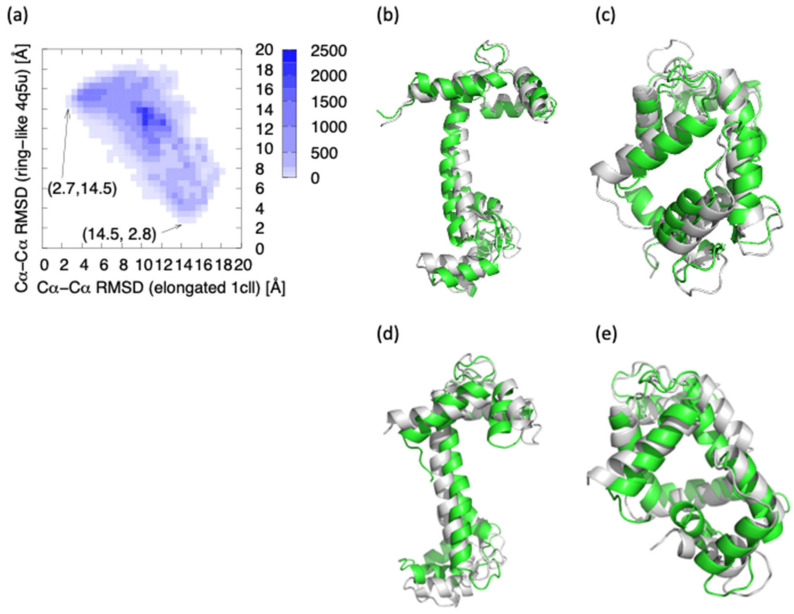
(**a**) The (unweighted) distribution of the structures obtained from MSDC-MD, as a function of Cα-Cα RMSD; the *x*- and *y*-axis are, respectively, Cα-Cα RMSD, with respect to the elongated structure (1cll) and the ring-like structure (4q5u). The total number of the samples is 252,168. (**b**) The elongated structure (white) and the most similar structure to the elongated structure obtained from the MSDC-MD simulation (green). (**c**) The ring-like structure (white) and the most similar structure to the ring-like structure obtained from the MSDC-MD simulation (green). (**d**) The elongated structure (white) and the representative structure around the elongated structure ($20\leq v_{1}\leq 50$ and $-20\leq v_{2}\leq 10$, green, RMSD = 3.2 Å). (**e**) The ring-like structure (white) and the representative structure around the ring-like structure ($-150\leq v_{1}\leq -120$ and $0\leq v_{2}\leq 30$, green, RMSD = 2.8 Å).

In order to confirm whether statistically sufficient samples were obtained or not, the RMSD, with respect to the elongated structure and the ring-like structure, were calculated (Figure 4a). It can be seen from the figure that the smallest Cα-Cα RMSD, with respect to the elongated structure and the ring-like structure, are, respectively, 2.7 Å and 2.8 Å. Considering that a Cα distance between adjacent amino acids is about 3.8 Å, the MSDC-MD has succeeded in sampling structures similar enough to the elongated structure or the ring-like structure. Indeed, when the structures of the minimum RMSD and representative structures obtained from MSDC-MD are superimposed on the elongated structure and the ring-like structure, it can be seen that they coincided well with each other (Figure 4b–e); the RMSD of the representative structure around the elongated and ring-like structure, with respect to the elongated and ring-like structure, was, respectively, 3.2 Å and 2.8 Å. Then, it can be said that the shape of the FEL around the elongated structure and the ring-like structure in Figure 3 is not due to a sampling error. Since the elongated structure was obtained from X-ray crystallography, the location of the elongated structure is away from the region where many NMR structures are located, which would reflect differences in experimental conditions. On the other hand, the ring-like structure is probably intrinsically unstable, and it is one that can only be stabilized by forming a complex.

**Figure 5:**
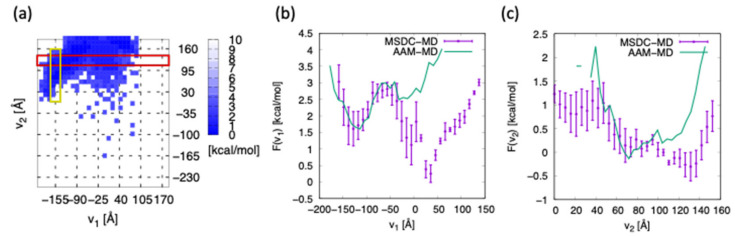
(**a**) FEL obtained from the conventional AAM-MD of 5 μs initiated at the elongated structure. (**b**) One-dimensional FEL obtained from the red rectangle in (**a**) as a function of $v_{1}$. (**c**) One-dimensional FEL obtained from the orange rectangle in (**a**) as a function of $v_{2}$.

To validate the correctness of the FEL, the FEL obtained by 5 μs conventional AAM-MD starting from an elongated structure is shown in Figure 5a. Further, the comparisons of one-dimensional FELs are shown in Figure 5b,c. The FEL shown in Figure 5b was obtained from averaging the two-dimensional FEL on the rectangle region from (−210 Å, 110 Å) to (190 Å, 140 Å) along the $v_{2}$-axis (red rectangle in Figure 5a). Similarly, the FEL shown in Figure 5c was obtained from averaging the two-dimensional FEL on the rectangle region from (170 Å, 0 Å) to (140 Å, 160 Å) along the $v_{1}$-axis (orange rectangle in Figure 5a). The position of the averaging area was shifted by ±10 Å, but the qualitative trend of the graph did not change.

It can be seen from Figure 5b that both AAM-MD and MSDC-MD sampled from the left basin located around $v_{1}$ = −130 Å. The shape of FELs roughly coincided with each other only around the left basin. However, AAM-MD failed in sampling above $v_{1}$= −50 Å, whereas MSDC-MD succeeded in finding a new basin around $v_{1}$ = 50 Å. This basin corresponds to the broad basin around ($v_{1},v_{2}$) = (50 Å, 100 Å), as shown in Figure 3, but AAM-MD does not detect the basin, as shown in Figure 5a. Therefore the FEL of AAM-MD at the right basin is shallower than that of MSDC-MD. AAM-MD, as shown in Figure 5c, could only sample a range of 40 Å < $v_{2}\;$<100 Å, while MSDC-MD could sample a much wider range of 0 Å < $v_{2}\;$< 160 Å.

**Figure 6:**
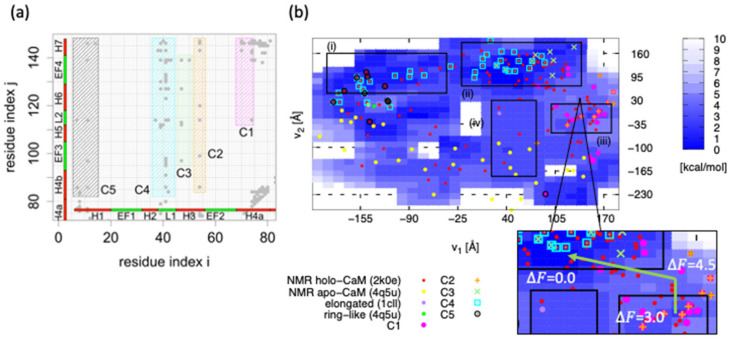
(**a**) The inter-domain contact probabilities whose contact ratio in holo-CaM is larger than that of chimera structures. The interactions can be divided into five groups (C1–C5). (**b**) The entire free-energy landscape obtained from MSDC-MD. The x- and y-axis are, respectively, $v_{1}$ and $v_{2}$. The red points show the NMR structures (PDB ID 2k0e), the orange points show the apo-NMR structure (1dmo), and the green and purple points, respectively, show the elongated structure (PDB ID 1cll) and the ring-like structure (PDB ID 4q5u). C1–C5 forming structures are, respectively, indicated as the pink point, orange cross, green cross, cyan square, and black circle. In the subset, the free-energy values relative to the region (iii) are shown in kilocalories per mole.

In our previous study [64], in order to identify the allosteric interactions among holo-CaM, the holo-CaM NMR structures (PDB ID: 1dmo) were compared to the “chimera” structure; the chimera structure was made by combining the linker region of the apo-CaM structures and the N- and C-lobe of holo-elongated CaM (PDB ID: 1cll). From the study, the interactions that are included in the holo-CaM NMR structures, but not in the chimera structures, had been identified as the contact probability (Figure 6a). Presumably, these interactions include allosteric interactions.

In order to investigate the relationship between the inter-domain interactions and the positions on the FEL, the inter-domain interactions are divided into five groups (C1–C5; Figure 6a), and the structures that have C1–C5 interactions were marked on the FEL (Figure 6b). As shown in Figure 6b, the structures forming C1–5 are projected following their own patterns instead of being projected randomly, indicating that the C1–5 interactions play essential roles in regulating the inter-domain motion. From Figure 6b, most NMR data are distributed (i) in the region around ($v_{1},v_{2}$) = (−140, 50), (ii) the region around (50, 90) and (iii) the region around (140, −30). Similarly, the FEL basins are in (i), (ii) and (iii), where the lowest point of the FEL is included in (ii), and the C3, C4 forming structures are populated. On the other hand, (iv) region (20 < $v_{1}\;$< 70, −200 <$\;v_{2}\;$< 50) is a broad and stable region, and the elongated structures are included here.

Since the distribution of NMR data of apo-CaM is basically distributed on the lower part of the FEL, the process of the initial structural change from apo-CaM to holo-CaM would correspond to the path on the FEL from the lower to the most stable point in (ii). The most probable path, in this regard, is the one passing through the region (iv). The reason why the experimentally famous elongated structure is actually included in this region may be the result of X-ray crystallography capturing the structural change from apo-CaM to holo-CaM upon Ca^2+^ binding. Since the lowest point of the FEL is located at point (ii), the ring-like structure is not the most stable state for monomeric holo-CaM. However, the FEL basin around the ring-like structure indicates that even the monomeric holo-CaM has interactions (C4, C5) to form the ring-like structure. Around the (iii) region, inter-domain interactions (C1, C2) are formed, but as the FEL barrier exits between region (ii) and (iii), as shown in the subset of Figure 6b, the difference of the relative free-energy to region (iii) (ΔΔF), between region (iii) and the saddle point of the barrier, is 1.5 kcal/mol, and ΔΔF between the saddle point and region (ii) is 4.5 kcal/mol. On the other hand, no clear barrier was found between region (iii) and (iv). Therefore, the structures that once went to region (iii) would back region (iv) against the barrier, while the inter-domain interactions formed in region (iii) are dissociated, in order to take an elongated structure. Then, if mutations that decrease the FEL barrier are generated, the mutated CaM may change its structure in a shorter time.

However, based on the amount of NMR data and the distribution of FELs, it is reasonable to assume that the structural changes occur in the order (iv), (ii), and (i) at this stage. Although the structural change of the individual AAM-MDs in the MSDC-MD is virtual behavior under fictitious confinement potentials, the overall FEL can be quickly calculated by reweighting the individual free energies. In this case, we compared the FEL of 200 ns MSDC-MD with that of the 5 μs, and the 200 ns MSDC-MD was able to calculate the FEL that is clearly broader and consistent with the NMR structure distribution. Furthermore, since the AAM-MDs in the MSDC-MD do not need to communicate with each other, it is very compatible with parallel computing. We can then conclude that the MSDC-MD is an excellent method for FEL analyses in recent situations where parallel computing is commonly used.

## 4. Conclusions

In our study, we applied a multi-scale simulation method that divides the conformation space into small areas to enable us to perform a well-equilibrated MD simulation, named ‘MSDC-MD’, to the free-energy analysis of CaM. In order to investigate the efficiency and accuracy, MSDC-MD simulation of CaM was performed. Since Komeiji et al. in 2002, CaM has been a good example to demonstrate the computational method, because it exhibits large conformational changes including domain rotation and translation in the Ca^2+^-signal transduction process. As the process takes nearly 1 ms, conventional MD simulations have failed to sufficiently sample wide conformational regions of CaM.

MSDC-MD succeeded in providing the overall conformational change of the CaM on the FEL yield by $v_{1}$ and $v_{2}$; the conformational samples obtained from MSDC-MD sufficiently covered the NMR and X-ray crystal structures indicating the efficiency of MSDC-MD. Furthermore, the MSDC-MD structures include samples similar to the famous elongated structure (PDB ID 1cll, RMSD = 2.8 Å at minimum) and ring-like structure (PDB ID 4q5u, RMSD = 2.7 Å at minimum), as shown in Figure 2. As the Cα- Cα distance connected by covalent bonds is about 3.8 Å, we conclude that the MSDC-MD succeeded in sampling the important structures, even though we started the simulations at different conformations.

The accuracy of the MSDC-MD was also investigated by comparing the FELs obtained by MSDC-MD and the conventional MD. Although a long ordinary MD simulation of 5 μs was performed, the conventional MD only gave left-upper region of FEL on the $v_{1}$, $v_{2}$ space. Then, one-dimensional FEL was calculated to compare the shape of FELs. The FEL obtained by the conventional MD on $v_{1}$ detected only the left basin, and it was trapped there until the end of the MD simulation. On the other hand, MSDC-MD can detect not only the left basin, but also another basin that is much deeper than the left one. Compared with the shape of the left basin, the shapes coincided with each other within the statistical error, which indicates that the MSDC-MD sufficiently reproduces the same entire FEL as the ordinary MD simulation, if the ordinary MD simulations of the millisecond time scale could be performed. The comparison of FEL on $v_{2}$ also demonstrated that the shape of FELs coincided with each other within the statistical error at the bottom of the ordinary MD’s FEL.

The FEL analysis provided the picture of how conformational changes occur on the FEL, and the important interactions on each stage of conformational change (C1–5) were linked to the FEL. The interactions C1–5 were defined to analyze the NMR structures in our previous study [64]. From the distribution of the NMR structures only, one might think that conformational change would occur along a path (iii) → (ii) → (i) on the upper region. However, the MSDC-MD’s FEL shows that the free-energy barrier between region (ii) and (iii), leads to the structures in region (iii) going back to region (iv). From the viewpoint of interactions, interactions C1–2 formed in region (iii) would be dissociated to form the elongated structure. Then, the process of conformational change on the FEL would occur along a path (iv) → (ii) → (i) when CaMBD is present. Such detailed information could not only be obtained from the experimental data, and the FEL analysis is necessary to complement the experimental data and investigate the biomolecular system more deeply.

## Data Availability

The data presented in this study are available on request from the corresponding author.
